# Dynamic expression of Dab2 in the mouse embryonic central nervous system

**DOI:** 10.1186/1471-213X-8-76

**Published:** 2008-08-04

**Authors:** Kwok-Kuen Cheung, Samuel C Mok, Payam Rezaie, Wood Yee Chan

**Affiliations:** 1 Department of Anatomy, Faculty of Medicine, The Chinese University of Hong Kong, PR China; 2 Department of Rehabilitation Sciences, The Hong Kong Polytechnic University, Hung Hom, Hong Kong, PR China; 3 Department of Gynecologic Oncology University of Texas M.D. Anderson Cancer Center T4.3908, 1515 Holcombe Boulevard Houston, TX 77030, USA; 4 Neuropathology Research Laboratory, Department of Life Sciences, Faculty of Science, The Open University, Walton Hall, Milton Keynes, MK7 6AA, UK; 5 Department of Neuroscience, Institute of Psychiatry, King's College London, DeCrespigny Park, London SE5 8AF, UK

## Abstract

**Background:**

Dab2, one of two mammalian orthologs of *Drosophila Disabled*, has been shown to be involved in cell positioning and formation of visceral endoderm during mouse embryogenesis, but its role in neuronal development is not yet fully understood. In this report, we have examined the localization of the Dab2 protein in the mouse embryonic central nervous system (CNS) at different developmental stages.

**Results:**

Dab2 protein was transiently expressed in rhombomeres 5 and 6 of the developing hindbrain between E8.5 and E11.5, and in the floor plate of the neural tube from E9.5 to E12.5, following which it was no longer detectable within these regions. Dab2 protein was also identified within circumventricular organs including the choroid plexus, subcommissural organ and pineal gland during their early development. While Dab2 was still strongly expressed in the adult choroid plexus, immunoreactivity within the subcommissural organ and pineal gland was lost after birth. In addition, Dab2 was transiently expressed within a subpopulation of Iba1-positive mononuclear phagocytes (including presumed microglial progenitors) within the neural tube from E10.0 and was lost by E14.5. Dab2 was separately localized to Iba1 positive cells from E9.5 and subsequently to F4/80 positive cells (mature macrophage/myeloid-derived dendritic cells) positioned outside the neural tube from E12.5 onwards, implicating Dab2 expression in early cells of the mononuclear phagocyte lineage. Dab2 did not co-localize with the pan-neuronal marker PGP9.5 at any developmental stage, suggesting that Dab2 positive cells in the developing CNS are unlikely to be differentiating neurons.

**Conclusion:**

This is the first study to demonstrate the dynamic spatiotemporal expression of Dab2 protein within the CNS during development.

## Background

Dab2 was first identified as DOC-2 which is *d*ifferentially expressed in *o*varian *c*arcinoma and cell lines [1]. It was later characterized as a tumor suppressor in various types of tumors [2-7]. Subsequent studies indicated Dab2 as one of two mammalian orthologs of the *Drosophila Disabled *(dDab) [8-11], a family member of the Disabled proteins involved in neurogenesis, oncogenesis, cellular signaling and embryonic development. Three different spliced forms of Dab2 cDNAs encoding variants p96, p93 and p67 were previously identified from a mouse macrophage library [12]. Of these, p96 was isolated as a mitogenic responsive phosphoprotein with possible function in the macrophage CSF-1 signal transduction pathway [12,13]. The complete genomic DNA sequence of the mouse Dab2 gene and its chromosomal localization at 15A2 were reported [14].

Sequence analyses [1,11,14] indicated Dab2 as an adaptor protein involved in signal transduction. The N-terminus of the protein contains a highly conserved phosphotyrosine interacting domain (PID or PTB) which interacts with proteins phosphorylated on tyrosine residue. Within its C-terminus, the proline-rich domain exhibits multiple SH3 binding motifs capable of interacting with SH3-containing signaling molecules such as Grb2. Signaling pathways involving Dab2 have not been fully characterized. However, there is evidence to suggest that Dab2 is an essential component of the transforming growth factor β (TGFβ) signaling pathway (involved in transmission of TGFβ signaling from TGFβ receptors to the Smad family of transcriptional activators) [15]. Dab2 has also been shown to act as a regulator of the Ras/mitogen-activated protein kinases (MAPK) pathway [16,17], Src activity [7] and canonical Wnt/β-catenin-induced signaling [18].

Considering that molecular interactions through such signaling pathways are important for normal growth and differentiation of embryonic structures, it has been proposed that Dab2 may also be involved in a number of different developmental processes [19]. For example, expression of Dab2 is upregulated upon retinoic acid treatment [20] or during the normal differentiation of F9 cells, a multipotent embryonic carcinoma cell line [21], into primitive endoderm-like cells [22]. Cho *et al*. also showed that the temporal expression pattern of the murine Dab2 gene coincided with the initiation pattern of retinoic acid synthesis in embryonic mice [20]. Furthermore, gene targeting studies have demonstrated that Dab2 is a direct and specific downstream target of the zinc finger transcription factor GATA-6 in the visceral endoderm [23]. In the absence of Dab2, the visceral endoderm becomes disorganized, resulting in growth failure of the inner cell mass. The Dab2-deficient embryo then fails to gastrulate and ceases development at around embryonic day 6.0 to 6.5 [19,23,24]. In addition to the visceral endoderm, Dab2 mRNA is also expressed in the branchial arch mesenchyme and septum transversum during development [23]. A recent study showed that Dab2 is required for TGFβ-induced epithelial-to-mesenchymal transition [25], which is an important multi-step process necessary for the proper development of many early embryonic structures including the neural crest, the primitive streak and somites [26-28].

Another mammalian ortholog of the *Drosophila Disabled *is *Dab1*, a related gene which also contains PID in its N-terminus. It has already been demonstrated that the Dab1 protein is important in regulating the positioning of migrating neurons during mammalian neurogenesis [29-31]. Mouse Dab1 is expressed in cortical neurons, and targeted disruption of Dab1 resulted in abnormal neuronal layering in the developing cerebral cortex and cerebellum, together with an indistinct dentate gyrus and CA1 and CA3 regions of the hippocampus [29]. Such defects partly resulted from a failure of Dab1^-/- ^neurons to detach from radial glia during the late stage of neuronal migration [30]. Despite the functional similarity of Dab1 and Dab2 proteins in cell positioning during development, it is not known whether Dab2 also play key roles during the development of the embryonic central nervous system (CNS), although it has recently been shown that Dab2 inhibits NGF-mediated neurite outgrowth *in vitro *[32]. Whether the expression of Dab2 is regulated in a developmental fashion has not been determined previously. Therefore in this investigation our primary aim was to characterize the spatiotemporal patterns of expression and the cellular localization of Dab2 protein within the embryonic murine CNS as an initial step towards further understanding the developmental roles of Dab2.

## Results

### Dab2 immunoreactivity in the floor plate and rhombomeres

**Figure 1:**
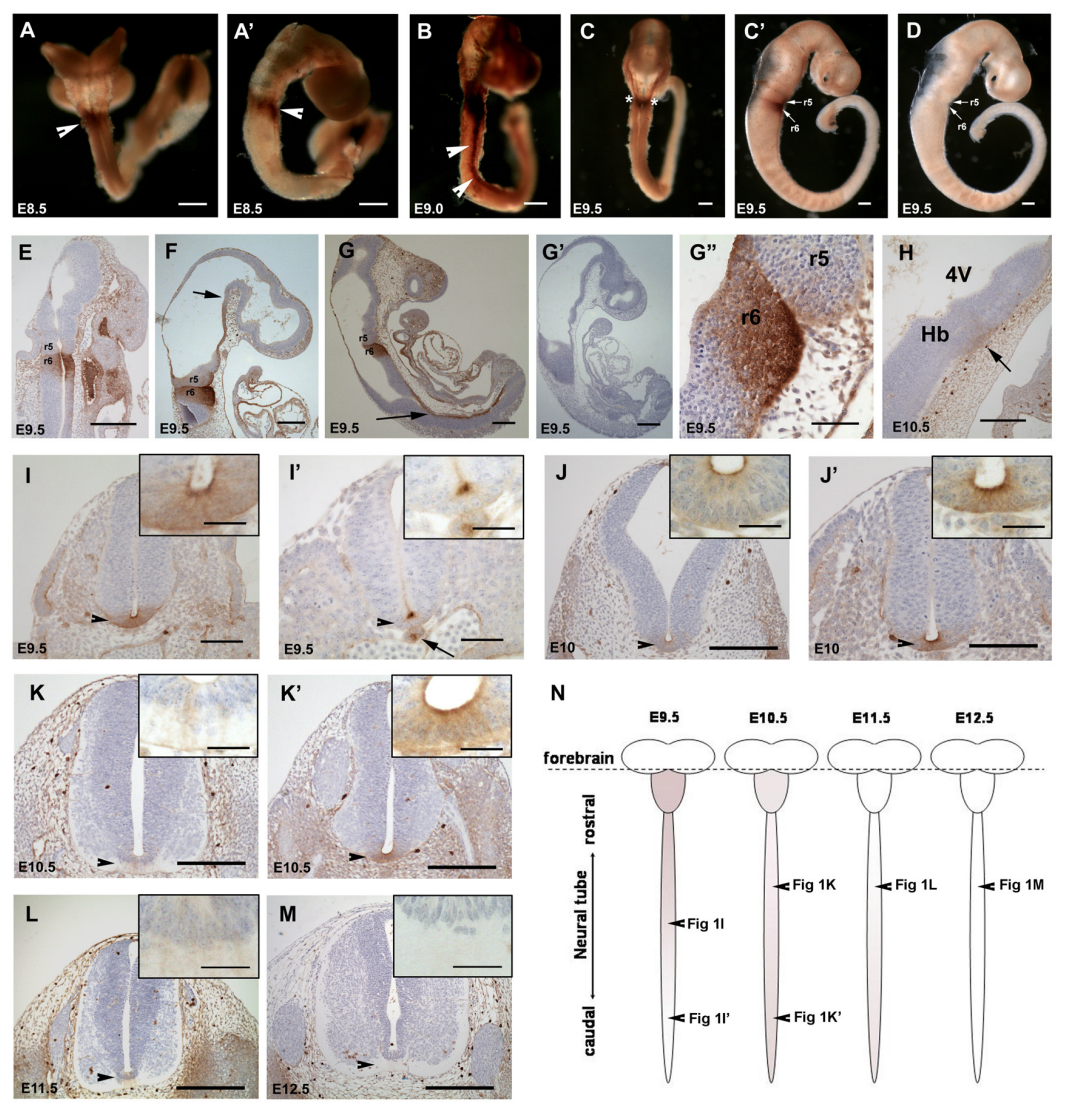
**Dab2 immunoreactivity in the neural tube**. In whole mount preparations (A-D), tissues surrounding the neural tube, including the otic vesicle (asterisks in C) had been removed by microdissection. At E8.5, the neural tube (A: dorsal view; A': lateral view) already shows Dab2 expression in rhombomeres 5 and 6 (arrowheads). At E9.0 (B: lateral view) and E9.5 (C: dorsal view; C': lateral view), expression within rhombomeres 5 and 6 (r5 and r6, arrows in C') remains strong, and spreads along the floor plate of the neural tube (arrowheads in B). The lack of immunoreactivity in the pre-adsorption control (D) confirms the specificity of the Dab2 immunoreaction. An oblique section (E) and longitudinal sections (F, G, G") through E9.5 embryos demonstrate strong Dab2 expression in the whole span of r6 and weak immunoreactivity in the ventral part of r5 (G" shows the high magnification of the immunoreactive r5 and r6 in G). The pre-adsorption control section (G') adjacent to the section shown in G does not exhibit any immunoreactivity. The ventral expression of Dab2 in the floor plate of the neural tube extends rostrocaudally from the mesencephalic flexure (arrow, F) to the caudal spinal cord (arrow, G). At E10.5, Dab2 immunoreactivity becomes weak in r6 (arrow, H) (Hb; hindbrain; 4V: fourth ventricle) and absent in r5. Dab2 immunoreactivity along the floor plate of the spinal cord (arrowheads, I to M) is also observed in transverse sections through the rostral (I, J, K, L, M) and caudal spinal cord (I', J', K') at different developmental stage. Also note the weak Dab2 staining in the notochord (arrow, I'). Insets in I to M represent the high magnification of the floor plate shown in corresponding figures. The schematic diagram (N) summarizes changes in Dab2 expression along the floor plate of the neural tube from E9.5 to E12.5. The dotted line indicates the axial level of the mesencephalic flexure and the shading represents Dab2 immunoreactivity observed in the floor plate of the neural tube. Arrowheads in N indicate regions of the neural tube from which sections shown in I, I', K, K', L, M were taken. Note that the onset and down-regulation of Dab2 expression follow a rostral-to-caudal sequence. Scale bar: 100 μm for I; 50 μm for G' and I', 25 μm for insets of I-L, and 200 μm for other panels.

Dab2 immunoreactivity was first observed in the neuroepithelium of the prospective hindbrain region at E8.5 (8–10 somites stage) (Fig 1A, A'). The expression was observed as a small positive band near the caudal margin of the closing anterior neuropore, corresponding to the position of rhombomeres 5 and 6 (Fig 1A, A'). This was regionally restricted, as other regions of the neuroepithelium lacked Dab2 expression. At E9.0 and E9.5, the neuroepithelium of rhombomere 6 was strongly Dab2 positive in its entire extent (Fig 1B, C, C', E, F), whereas only the ventral part of rhombomere 5 showed immunoreactivity (Fig 1F, G, G"). In addition to rhombomeres 5 and 6, Dab2 was also expressed in a narrow strip of the ventral neural tube extending from the mesencephalic flexure down to the spinal neural tube (Fig 1B, F, G). Sections through the spinal neural tube revealed Dab2 expression restricted to the floor plate (Fig 1I, I'). Dab2 expression in the floor plate was initially detected in the rostral part of the spinal cord and gradually shifted in a rostral-to-caudal manner to the caudal spinal cord (Fig 1N, compare Fig 1I with Fig 1I'). Weak Dab2 immunoreactivity was also detected in the caudal part of the notochord (Fig 1I'). By E10.5, the Dab2 protein expression was significantly down-regulated in these rhombomeres (Fig 1H) and became undetectable by E11.5. Likewise, Dab2 expression was progressively down-regulated in the floor plate of the neural tube from E10.5 onwards. Down-regulated expression of Dab2 in the floor plate, also occurred in a rostral-to-caudal direction along the spinal cord (Fig 1N, also compare Fig 1I–K with Fig 1I'–K'). Dab2 immunoreactivity in the floor plate became undetectable by E12.5 (Fig 1M). Negative controls did not show any immunoreactive signals (Fig 1D, G').

### Dab2 immunoreactivity in the developing choroid plexus

**Figure 2:**
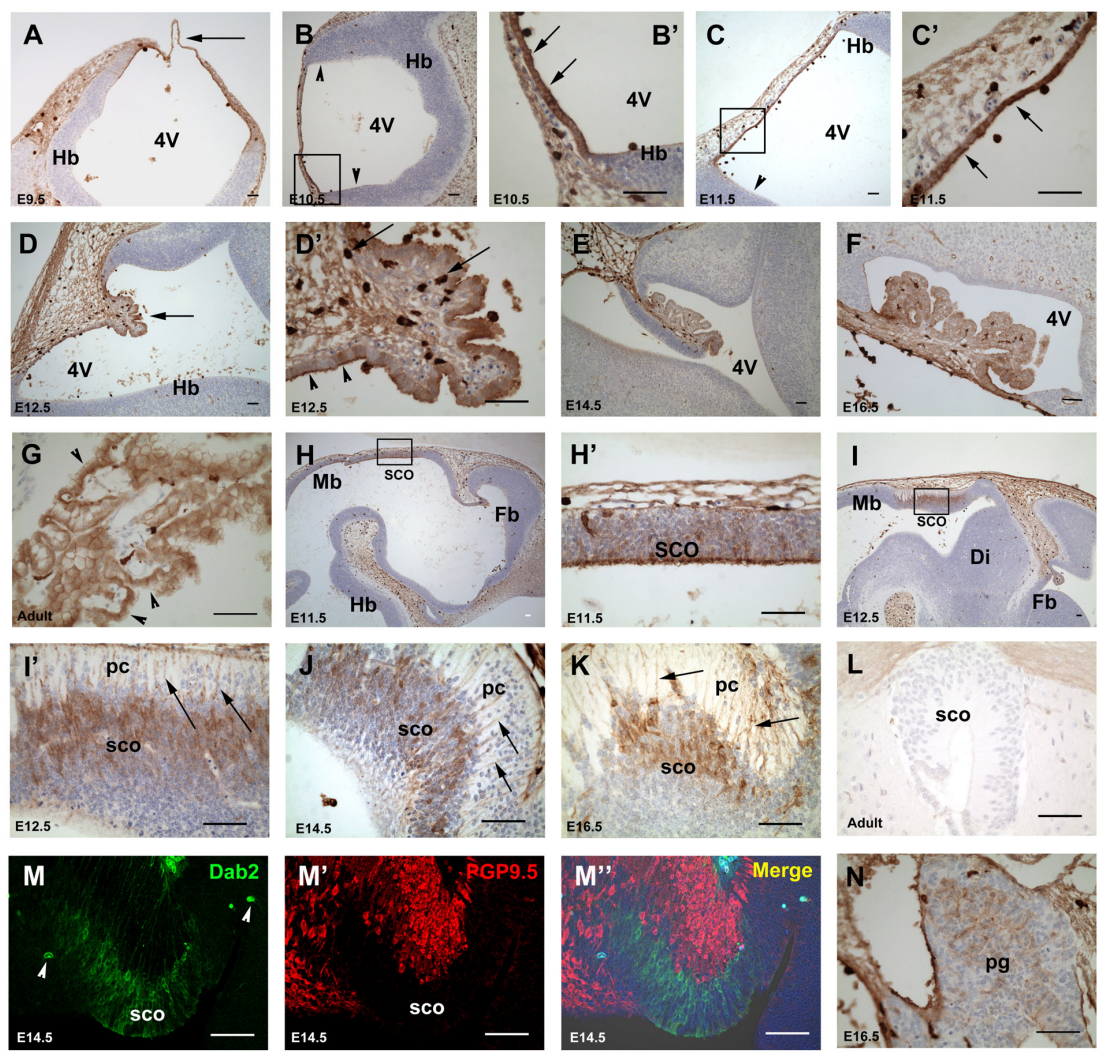
**Dab2 immunoreactivity in the developing choroid plexus, subcommissural organ and pineal gland**. Sections through the hindbrain (Hb) at stages from E9.5 to E11.5 (A to C; B' and C' represent the high magnification of the boxes in B and C, respectively) show Dab2 expression in the roof plate of the hindbrain (arrows) and on scattered round cells (mononuclear phagocytes). Note that the adjacent hindbrain neuroepithelium is negative for Dab2 staining (arrowheads in B, C). Sections through the hindbrain (Hb) at stages from E12.5 to adulthood (D to G, D' is the higher magnification of the choroid plexus shown in D) show Dab2 staining in the choroid plexus (e.g. arrow in D) of the fourth ventricle (4V). Note the stronger Dab2 staining in the luminal (apical) surface of the epithelial cells as compared to the corresponding basal surface in embryos (arrowheads in D), but however, in the adult choroid plexus, the basal surface shows stronger Dab2 staining than the luminal (apical) surface of the epithelial cells (arrowheads in G). Also note the scattered and strongly Dab2 positive macrophages (arrows in D'). Sagittal sections show Dab2 staining in the subcommissural organ along the dorsal midline at different stages (H to K; H' and I' represent the higher magnification of the boxes in H and I, respectively). Note that Dab2 protein is expressed in the neuroepithelium of the subcommissural organ (sco) located in the roof plate of the diencephalon (Di). It lies underneath the posterior commissure (pc), anterior to the midbrain (Mb) (I', J, K). Also note the thin processes (arrows in I', J, K), which are also Dab2 positive, extending from the subcommissural organ into the posterior commissure. Coronal section (L) shows absence of Dab2 staining in the adult subcommissural organ. Double labeling of Dab2 (green, M) with PGP9.5 (red, M') shows that they are not co-localized (M"). Arrowheads in M indicate autofluorescence in blood cells. The sagittal section (N) shows Dab2 staining in the developing pineal gland (pg) along the dorsal midline at E16.5. Fb: forebrain; Mb: midbrain; Hb: hindbrain. Di: diencephalon. 4V: 4^th ^ventricle; pc: posterior commissure; sco: subcommissural organ; pg: pineal gland. Scale bar: 50 μm.

Dab2 protein was detected in the roof plate of the hindbrain between E9.5 and E11.5 (Fig 2A, B, B', C, C'). At E12.5, the developing choroid plexus of the lateral ventricles and the fourth ventricle (Fig 2D, D') were Dab2 immunoreactive. The choroid plexus of the third ventricle, which developed slightly later, was also positive for Dab2 (data not shown). However, the neuroepithelium adjacent to the choroid plexus was clearly negative and a distinct boundary between the immunoreactive choroid plexus and the adjacent neuroepithelium which lacked staining, was easily discernible (Fig 2B–D). The entire choroid plexus, including most of the epithelial cells that lined the ventricles and their underlying mesenchyme, was Dab2 positive (Fig 2D-F, 2D'). More intense expression was associated with the apical aspect (or luminal surface, and associated microvilli) as compared to the basal aspect of epithelial cells (Fig 2D'). The choroid plexus continued to express the Dab2 protein in adulthood, and consequently, became the most prominent Dab2 positive structure in the adult CNS (Fig 2G), with the exception that the intensity and localization of Dab2 expression was reversed: in contrast to the embryonic choroid plexus, which showed strong Dab2 immunoreactivity on the luminal (apical) side of the epithelium (Fig 2D'), the luminal surface of the epithelial cells in the adult choroid plexus showed weaker immunoreactivity compared to the basal surface (Fig 2G).

### Dab2 immunoreactivity within the developing subcommissural organ and pineal gland

The presumptive subcommissural organ (SCO) develops from the region of the roof plate of the diencephalon just anterior to the mesencephalon (Fig 2H, H'). As development progresses, the SCO neuroepithelium, which lies underneath (ventral to) the posterior commissure, became morphologically more distinct from the adjacent neuroepithelium (Fig 2I–K). Dab2 immunoreactive cells were detected in the developing SCO at E11.5 and the expression became progressively more prominent (Fig 2H–K, 2H'–I'), particularly in the cell layers immediately below the posterior commissure and also in the thin processes extending from the SCO to the posterior commissure (Fig 2I', 2J–K). The ventricular aspect of the SCO however showed only weak Dab2 immunoreactivity (Fig 2I', 2J–K). Double immunofluorescent labeling of Dab2 and PGP9.5, a pan-neuronal marker, was performed in order to determine whether Dab2 was expressed in neurons or nerve fibers that were closely associated with the SCO. In embryos at late stages (E14.5–E16.5) the SCO neuroepithelium was Dab2 positive but negative for PGP9.5, whereas the posterior commissure above the SCO was PGP9.5 positive but negative for Dab2 (Fig 2M–M"), indicating that Dab2-immunoreactive cells were unlikely to represent differentiating neurons. Dab2 expression was persistently detected in the SCO during prenatal development but was not found in the adult (Fig 2L). The pineal gland additionally showed Dab2 immunoreactivity at E16.5 (Fig 2N) but Dab2 was not detectable in the adult pineal gland (data not shown).

### Dab2 immunoreactivity in developing mononuclear phagocytes (fetal macrophages and microglial progenitors)

#### Within the developing neural tube

**Figure 3:**
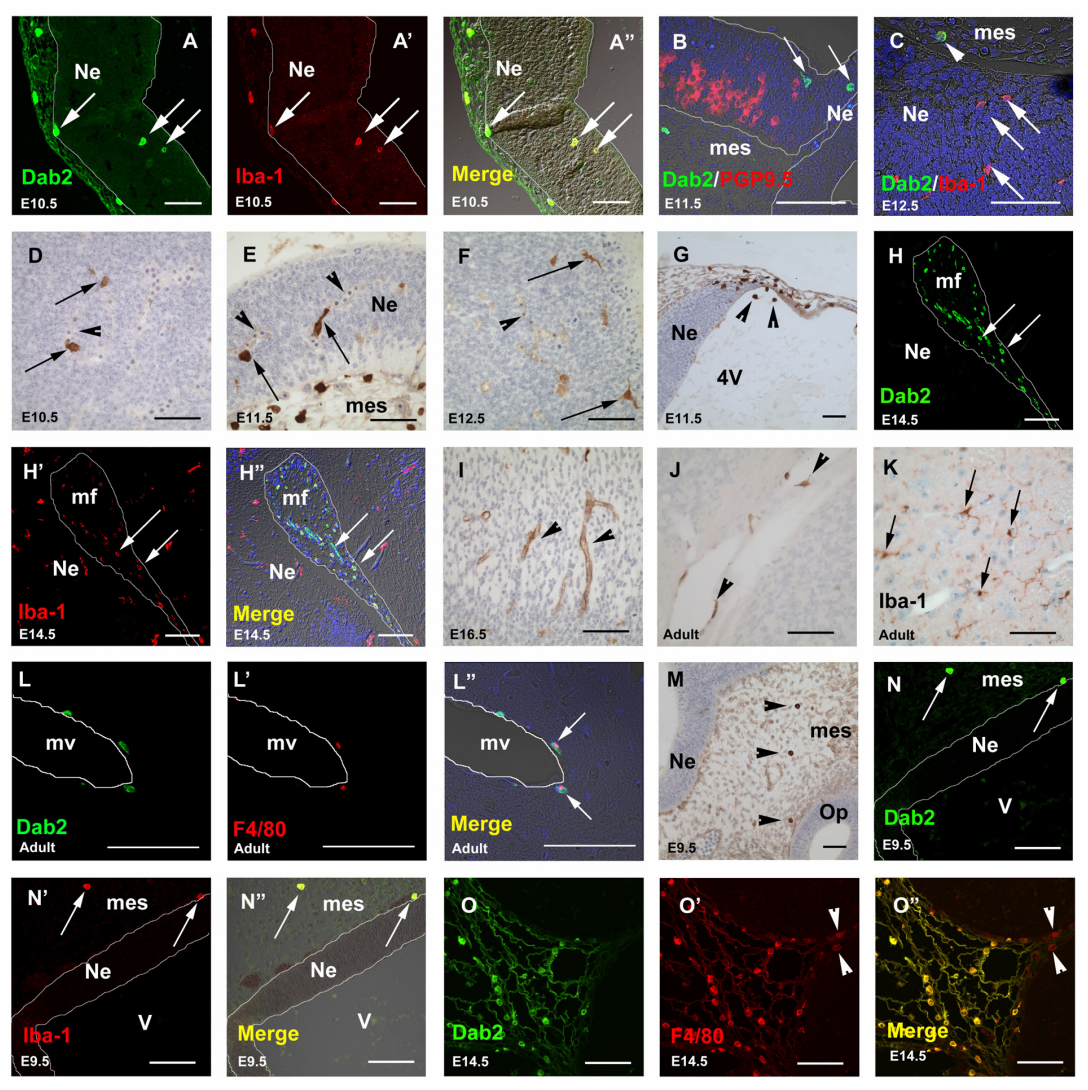
**Dab2 immunoreactivity in mononuclear phagocytes**. A-A": Double labeling (immunofluorescence staining) on a sagittal section through an E10.5 embryos shows co-localization of Dab2 (green, arrows in A) and Iba1 (red, arrows in A') in mononuclear phagocytes (yellow, arrows in A") within the neural tube (Ne, white outline), and in fetal macrophages outside the neural tube. B: Double labeling shows that Dab2 immunoreactivity (green, indicated by arrows) does not overlap with PGP9.5 immunoreactivity (red) within the neural tube (white outline) in a sagittal section through an E11.5 embryo. C: Double labeling at E12.5 shows Iba1^+^/Dab2^- ^microglial progenitors (arrows, red) within the neural tube (Ne) and Iba1^+^/Dab2^+ ^fetal macrophages (arrowhead) in the adjacent mesenchyme (mes).D-F: Sagittal sections show that Dab2^+ ^cells (arrows) are often closely apposed to blood vessels (arrowheads) within the neural tube at E10.5 (D) and E11.5 (E), but more scattered by E12.5 (F). G: Some Dab2^+ ^cells (arrowheads) are also observed closely associated with the roof of the fourth ventricle (4V). H-H": Double labeling for Dab2 (green, H) and Iba1 (red, H') at E14.5 shows that Dab2 is only expressed in Iba1^+ ^fetal macrophages (arrows) in the mesenchyme (mf: mesencephalic flexure, white outline) outside the neural tube (Ne) but absent in Iba1^+ ^microglial progenitors within the neural tube. I: At E16.5, Dab2 expression is detected in the wall of blood microvessels (arrowheads), but Dab2^+ ^microglial progenitors are no longer detectable at this stage of development. J, K: Sections of an adult brain show Dab2^+ ^perivascular cells (arrowheads in J) and Iba1^+ ^microglia (arrows in K). L-L": A merged image (L") shows co-localization of F4/80 protein (red, L') in some (arrows in L") but not all of the Dab2^+ ^(green, L) cells lying on the wall of cerebral microvessels (mv, white outline). M: A sagittal section of an E9.5 embryo shows Dab2^+ ^cells in the cranial mensenchyme (mes) outside the neural tube (Ne). N-N": A merged image at E9.5 shows complete co-localization (yellow, brightfield, arrows in N") of Dab2 (green, arrows in N) and Iba1 (red, arrows in N') immunoreactivities in fetal macrophages outside the neural tube (Ne, white outline). O-O": A section through an E14.5 choroid plexus shows complete co-localization (yellow, O") of Dab2 (green, O) and F4/80 (red, O') immunoreactivities. Arrowheads in O' and O" indicate autofluorescence from red blood cells. DAPI counterstained in B, C, H", L". Scale bar: 50 μm.

Scattered but strongly Dab2-immunoreactive cells were detected within the neuroepithelium at E10.0 – E10.5 (Fig 3A). Double labeling with the Iba1 antibody (Fig 3A'), a mononuclear phagocyte marker expressed on cells of the macrophage and microglial cell lineages [33-35], showed that the Dab2 protein was co-expressed with Iba1 protein (Fig 3A"). Dab2 immunoreactive cells did not express PGP9.5, indicating that they were not early differentiating neurons (Fig 3B). Although all Dab2-positive cells showed Iba1 immunoreactivity, cells that were Iba1 positive but Dab2 negative (Iba1^+^/Dab2^-^) were also observed (Fig 3C). Iba1^+^/Dab2^+ ^cells resembled the early fetal macrophages previously reported in the developing CNS [35], several of which were found in the proximity of blood vessels at E10.5 (Fig 3D) and E11.5 (Fig 3E). However, their close relationship with blood vessels was lost at E12.5, at which time Iba1^+^/Dab2^+ ^cells were found dispersed throughout the neural tube (Fig 3F). Iba1^+^/Dab2^+ ^fetal macrophages were also frequently detected inside the ventricles, in regions close to the ventricular wall (Fig 3G). From E14.5 onwards, however, scattered Iba1^+^/Dab2^+ ^cells were no longer detected within the neural tube, leaving only Iba1^+^/Dab2^- ^cells (presumed to represent parenchymal microglial progenitors) within the neural tube (Fig 3H, H', H"). The endothelial lining of the brain microvasculature also expressed Dab2 from E11.5 onwards (Fig 3E, F, I). In the adult, however, Dab2 immunoreactivity persisted only in perivascular macrophages (also widely referred to as 'perivascular cells') associated with cerebral blood vessels, but was absent from the endothelial lining of these vessels (Fig 3J). Perivascular cell expression of Dab2 was prominent throughout the entire adult CNS. These cells were not uniform in shape but were either oval with small processes, or flat and elongated in shape (Fig 3J), as has been previously described [36,37]. Unlike embryonic CNS, where cells closely apposed to the microvasculature expressed both Iba1 and Dab2 protein, Dab2 positive perivascular cells in the adult brain did not appear to co-express Iba1 protein (Fig 3J–K). Instead, double immunofluorescent labeling revealed that some, but not all, of these Dab2 immunopositive perivascular cells co-expressed F4/80 antigen, indicating that they were of the macrophage lineage (mature macrophages) (Fig 3L, L', L").

#### Outside the developing neural tube

Scattered cells strongly positive for Dab2 were also detected within mesenchymal tissues outside the neural tube from E9.5 (Fig 3M), about half a day earlier than Iba1^+^/Dab2^+ ^cells inside the neural tube. These Dab2 positive cells were also Iba1 positive (Fig 3N, N', N"). The macrophage differentiation marker, F4/80 antigen, began to be expressed weakly in these fetal macrophages located outside the neural tube at E12.5 and became prominent from E14.5 onwards. Double immunofluorescence staining for Dab2 and F4/80 showed that all Dab2 positive cells were also F4/80 immunoreactive from E14.5 onwards (Fig 3O, O', O"). In contrast to the transient, developmentally-restricted expression of Dab2 protein associated with mononuclear cells (microglial progenitors) located inside the neural tube, Dab2 protein continued to be persistently expressed in macrophages throughout the body into adulthood (data not shown).

## Discussion

We have characterized in detail the expression pattern for Dab2 in the mouse embryonic central nervous system throughout development. Dynamic expression of Dab2 protein was detected in the neuroepithelium, blood vessels, circumventricular organs and mononuclear phagocytes (fetal macrophages, microglial progenitors), strongly suggesting that Dab2 is an important molecule during mammalian CNS and myeloid cell development. Unlike Dab1, which is expressed in cortical neurons, Dab2 expression is restricted to non-neuronal cells or immature neuroepithelial cells.

At E8.5, while signaling genes such as *Hoxa1*, *Hoxa2*, *Hoxb1*, *kr *and *Krox20 *are interacting to pattern the early hindbrain [38], Dab2 is expressed in presumptive rhombomeres 5 and 6 (r5-6). There are 7 to 8 rhombomeres, morphologically repeated compartments, transiently segregated along the anteroposterior axis of the vertebrate embryonic hindbrain. These compartments play pivotal roles in the specification and segregation of neurons and neural crest cells along the anteroposterior axis of the hindbrain [39]. It is well-established that genes with rhombomere-restricted expression (e.g. *Hox *genes) function to establish the identities of compartments [40]. Interestingly, it has been shown that Dab2 expression is significantly up-regulated in *Hoxa1 *knockout embryonic stem cells [41]. In the early hindbrain at E8.0 *Hoxa1 *is expressed in r4-6 and gradually restricted to r4 at E8.5 [38]. Such down-regulation correlates spatiotemporally with the onset of Dab2 expression within r5-6 at E8.5. A further understanding of the interactions between Dab2 and Hoxa1 will greatly enhance our knowledge of signaling cascades associated with hindbrain specification.

During development, dorsoventral patterning along the neural tube is controlled by the expression of *sonic hedgehog *(*shh*) [42]. *shh *is expressed by cells along the ventral midline of the embryonic forebrain, in the floor plate and the notochord of the hindbrain and spinal cord [43]. Since the encoded protein is in a secreted form, Shh protein expression displays a more diffuse pattern [43]. Dab2 protein expression appears to overlap with the region expressing *shh *mRNA in the floor plate and notochord at E9.5 but not the regions showing Shh protein [43]. Moreover, Dab2 protein expression is turned off much earlier in the floor plate (absent in E12.5) than for Shh in the ventral spinal cord at E15.5 [43]. The potential involvement of Dab2 in anteroposterior and dorsoventral patterning requires further investigation.

Dab2 protein was also detected within circumventricular organs including the choroid plexus, the subcommissural organ and the pineal gland. Dab2 may represent one of the earliest markers for the choroid plexus primordium in the roof plate of the hindbrain. Its persistent expression in the choroid plexus throughout life, however, indicates that Dab2 may not only be involved in the early formation of the choroid plexus but may also have a role in the transport of macromolecules across the epithelial cells of the choroid plexus to the cerebral ventricles. Evidence to support this hypothesis comes from studies on megalin, a well-known receptor for Dab2 [44]. Megalin mediates the transport of apolipoprotein J, Alzheimer's amyloid-beta peptide, leptin, insulin-like growth factor-I, and also regulates macromolecule transport from the cerebral microvessels and choroid plexus to cerebral ventricles, thereby maintaining homeostasis in cerebrospinal fluid (CSF), as well as exerting neuroprotective action [45-47]. In the absence of Dab2, the protein levels of megalin are reduced and the subcellular localization of megalin is altered [48]. The choroid plexus starts to secrete CSF very early during development [49], correlating well with the early expression of Dab2 protein. We found that Dab2 is also prominently expressed in microvilli on the luminal surface of the embryonic choroid epithelium, consistent with a role associated with macromolecule transport and secretory functions. However, the change observed in asymmetric expression between the luminal and the basal surface from embryonic to adult states (i.e. more prominent expression in the basal compared to luminal aspect in the adult) remains unclear.

Dab2 immunoreactivity was detected in another developing circumventricular organ, the subcommissural organ (SCO). The SCO develops very early during embryogenesis, representing one of the first secretory brain structures to differentiate [50]. The very early ontogenetic differentiation of SCO secretory cells is crucial for the smooth circulation of CSF secreted from the choroid plexus during early development. The present study shows that Dab2 is specifically expressed in the developing SCO primordium at an early stage (E11.5). Both the choroid plexus and the SCO are secretory organs that develop very early in embryos. However, unlike the early and persistent expression of Dab2 in the choroid plexus, the Dab2 protein appeared transiently in SCO-secretory cells, a phenomenon that may possibly be related to the morphogenesis of SCO. Similar to the SCO, the developing pineal gland also showed transient Dab2 expression in the present study. Dab2 expression in the developing pineal gland was not detected at or before E14.5 but became distinct by E16.5, the stage at which pinealocytes undergo rapid proliferation in rodents [51]. It is likely that Dab2 is expressed in developing pinealocytes, although we cannot rule out the possibility that interstitial cells also express the Dab2 protein. The functional significance of a transient expression of Dab2 in the developing pineal gland remains to be investigated.

Other than the expression in secretory epithelial cells, Dab2 was also detected in cells of the myeloid lineage, specifically mononuclear phagocytes. We have clearly shown that Dab2 is expressed at early developmental stages in mononuclear phagocytes (Iba1^+^/Dab2^+ ^cells) both outside the developing neural tube (from E9.5 onwards) and transiently *within *the neural tube (from E10.5 – E14.5). The expression of Dab2 (or p96) in mature macrophages is perhaps not surprising given its initial characterization from a macrophage cell line [11-13]. However, the functional roles played by Dab2 in lineage commitment and differentiation of myeloid-derived mononuclear phagocytes are only recently being investigated [52], and have clear implications for determining the lineage and differentiation also of microglia. ICSBP (Interferon Consensus Sequence Binding Protein; a member of the interferon regulatory factor family of transcription factors which acts as a regulatory switch in myeloid differentiation [52]), and the Ets transcription factor PU.1, are haematopoietic-restricted transcription factors critical for the development of haematopoietic cells [52]. PU.1 is an essential factor required for myeloid cell differentiation [35]. Both of these transcription factors bind to the Dab2 promoter, but whereas ICSBP represses Dab2 promoter transactivation, PU.1 induces it [52]. Significantly, ICSBP represses the PU.1-induced transactivation, thereby suppressing Dab2 expression. These data indicate that cross-talk between PU.1 and ICSBP may be crucial in regulating genes related to myeloid cell differentiation and cell functions. Taken together and in context with our current findings showing an increased and persistent expression of Dab2 in developing and adult macrophages compared to the lack of Dab2 in adult microglia, these results allude to differences in myeloid cell differentiation (and/or commitment) between macrophages and microglial cells [35] that need to be explored in greater depth.

Transient expression of Dab2 was detected in early Iba-1^+^cells located within the neural tube, in cells closely apposed to blood vessels and to the ventricular walls. These observations may imply that at least a subpopulation of early microglia (Iba1^+^/Dab2^+^) are derived from myeloid progenitors migrating into neural tube via microvessels, rather than derived *in situ *[35]. Downregulation, or indeed lack of Dab2 expression in subsequently developing microglia may represent an important event during the differentiation and maturation of these cells.

Dab2 acts as a signalling molecule in CSF-1/MCSF (colony stimulating factor-1/macrophage colony stimulating factor) signal transduction [12,13]. Microglia, unlike cells of the macrophage lineage, are not exclusively dependent on CSF-1 for cell survival and differentiation [53-56]. The lack of Dab2 protein expression in microglia (Iba1^+^/Dab2^-^) which persists throughout development (E12.5-14.5 onwards in this study), in adult CNS microglia at rest (this study), and even under traumatic injury [57], may reflect this lack of dependency on CSF-1/MCSF for differentiation and/or survival of these cells, which in turn points to differences in environmental trophic support for mononuclear phagocytes within the CNS.

Although Dab2 was not expressed in adult microglia, it was expressed in another mononuclear phagocytic cell type in the CNS, namely perivascular macrophages which co-expressed F4/80 antigen. The differential expression of Dab2 in mononuclear phagocytes (microglia, fetal and adult macrophages and perivascular macrophages) renders it a useful marker to further study the origin and responses of these cells.

## Conclusion

This is the first detailed report on the spatio-temporal expression of Dab2 during CNS development in mammals. Unlike Dab1, which is expressed in developing neuronal cells, Dab2 protein is dynamically expressed in various regions of the developing CNS, primarily restricted to non-neuronal cells including within circumventricular organs, in secretory cells, immature neuroepithelial cells, endothelial cells and in populations of mononuclear phagocytes (including fetal and perivascular macrophages), but lacking in neurons, neuroglial cells and in mature microglia. These findings are consistent with its expression pattern observed in the adult nervous system (this study and [57]), and in response to CNS injury [57] where Dab2 expression is primarily located to macrophages accumulating at the site of injury, but absent from neurons, neuroglia and microglia. Expression of Dab2 in polarized epithelial cells in developing circumventricular organs may be consistent with the well-documented functions of Dab2 in mediating endocytic trafficking of clathrin-coated vesicles [58,59], and in directing cell surface positioning [60]. Dab2 is further implicated in myeloid cell differentiation and macrophage development. Our findings highlight the need for further investigations into the biological functions of Dab2 in early CNS and myeloid cell development.

## Methods

### Experimental animals

Random-bred ICR mice were kept at a constant temperature of 23 ± 2°C under a 12-hr light: 12-hr dark cycle at the Laboratory Animal Services Centre of the Chinese University of Hong Kong. They were provided with food and water *ad libitum*. All experimental procedures involving the use of animals for experimental purposes were performed under licence from the Government of the Hong Kong SAR and endorsed by the Animal Experimentation Ethics Committee of the Chinese University of Hong Kong (Animal Ethics Approval Ref. No. 04/006/ERG). Female mice weighing 35–40 g were paired with a male mouse overnight. The noon of the plug day was designated as embryonic day 0.5 (E0.5) assuming that copulation occurred around midnight.

### Immunohistochemical staining and double immunofluorescence staining on tissue sections

Pregnant mice at different stages of pregnancy from E7.5 to E18.0 were sacrificed by cervical dislocation and uteri dissected open. For each developmental stage, at least 5–10 embryos were collected for analysis. Embryos were isolated, fixed in Bouin's fixative, and processed for wax sectioning. Sections of 7 μm thick were cut and mounted on Superfrost^® ^Plus microscopic slides (VWR Scientific). Localization of the Dab2 protein by immunohistochemical staining was performed using the monoclonal anti-p96/Dab2 antibody (1:400, Transduction Laboratories/BD Biosciences). The peptide used to raise this monoclonal antibody corresponds to amino acids at positions 31–45 at the N-terminal of the full-length murine p96/Dab2; a domain that is shared by the three known isoforms of Dab2 (namely p96, p93 and p67) in the mouse. Antigen retrieval was carried out by microwave treatment in 0.1 M citric acid buffer (pH 6.0). Immunostaining was performed with the standard ABC method using DAB (Sigma) as the chromogen [2,61]. Sections were counter-stained with hematoxylin, dehydrated in a graded series of alcohols, cleared in xylene and mounted in Permount (Fisher Scientific). Images were taken under a Zeiss microscope with a digital camera (Diagnostic instruments, USA). The specificity of p96/Dab2 was confirmed by (1) pre-adsorbing with the corresponding purified immunogen (BD Biosciences) for 6 hr at 4°C before adding to the sections, or (2) replacing the primary antibodies with PBS containing 5% normal horse serum. For double immunohistochemical localization, 4% paraformaldehyde fixed sections and antibodies to Dab2 (1:100, BD), Iba1 (1:75, Wako), F4/80 (1: 50, Serotec), PGP 9.5 (1: 800, Ultraclone) were used. Secondary antibodies used were Alexa Fluor-conjugated donkey antibodies against mouse, rabbit or rat (1:400, Invitrogen). Immunofluorescent images were captured using a confocal microscope (Olympus FluoView™-FV1000).

### Whole mount immunohistochemistry

The procedure was carried out as previously described [62]. Briefly, embryos were collected and fixed in Bouin's fixative. They were dehydrated with upgraded methanol and stored in pure methanol at -20°C until use. Embryos were then treated with 2% Triton X-100 in DMSO/methanol (4:1) overnight for permeabilization. They were washed in 0.5% Triton X-100 in PBS (PBT) and treated with 1% periodic acid overnight to block endogenous peroxidase activity. Non-specific binding was blocked with 5% non-fat dry milk in PBT (PBTM) before incubating the embryos at 4°C overnight with the monoclonal anti-p96 antibody (1:400) [19,24]. Signals were amplified with the standard ABC methods using DAB as chromogen. The entire neural tube was isolated using tungsten needles and brought to 80% glycerol in distilled water, through a graded series, to obtain transparency. Images were captured with an AxioCam (Zeiss) under a stereomicroscope (Zeiss).

## Authors' contributions

K–KC carried out most of the staining on developing mouse embryos, analyzed and helped interpret the staining patterns and drafted the manuscript. SCM conceived the study, helped design the protocol for immunohistochemical staining and helped draft part of the manuscript. PR participated in the design of Iba1-Dab2 and F4/80-Dab2 double staining, interpreted the observations concerning mononuclear phagocytes and helped draft part of the manuscript. WYC participated in the overall design of the entire study, coordinated different parts of the study, helped analyze the results and helped draft part of the manuscript. All authors read and approved the final manuscript.
